# Association of TRPS1 rs2737229 and TRIB1 rs2954029 Genetic Polymorphisms with Subclinical Atherosclerosis, LDL Cholesterol, and Glucose Levels

**DOI:** 10.3390/biology15070580

**Published:** 2026-04-05

**Authors:** Gilberto Vargas-Alarcón, Rosalinda Posadas-Sánchez, Oscar Pérez-Méndez, Marva Arellano-González, José Manuel Fragoso

**Affiliations:** 1 Research Direction, Instituto Nacional de Cardiología Ignacio Chávez, Mexico City 14080, Mexico; gvargas63@yahoo.com; 2 Department of Endocrinology, Instituto Nacional de Cardiología Ignacio Chávez, Mexico City 14080, Mexico; rossy_posadas_s@yahoo.it; 3 Department of Molecular Biology, Instituto Nacional de Cardiología Ignacio Chávez, Mexico City 14080, Mexico; opmendez@yahoo.com (O.P.-M.); marva_01@hotmail.com (M.A.-G.)

**Keywords:** subclinical atherosclerosis, transcriptional repressor GATA binding 1, Tribbles pseudokinase 1

## Abstract

Recent reports have shown that polymorphisms in the *TRPS1* and *TRIB1* genes are associated with plasma lipid levels and the risk of cardiovascular disease. This study evaluates the associations of *TRPS1* and *TRIB1* polymorphisms with subclinical atherosclerosis (SA) and plasma lipid levels in Mexican individuals. This study included 1406 Mexican mestizo individuals (417 individuals with SA and 989 healthy controls). Genotyping of *TRPS1* and *TRIB1* polymorphisms was performed using TaqMan assays in a real-time PCR system. To analyze whether these polymorphisms are associated with SA and plasma lipid levels, we used logistic regression (OR [95% CI]), adjusted for confounding factors. The *AA* genotype of the *TRPS1* rs2737229 *A*/*C* polymorphism showed a significant association with the risk of developing SA under multiple genetic models [codominant: OR = 1.61 (95% CI: 1.10–2.36), *p* = 0.048; recessive: OR = 1.42 (1.02–1.99), *p* = 0.039; additive: OR = 1.26 (1.05–1.53), *p* = 0.015]. Similarly, the *TT* genotype of the *TRIB1* rs2954029 *T*/*A* polymorphism was also significantly associated with the risk of developing SA [codominant: OR = 1.63 (1.10–2.43), *p* = 0.033; recessive: OR = 1.64 (1.13–2.37), *p* = 0.009]. In a sub-analysis of SA individuals, individuals with homozygous *AA* for the *TRPS1* rs2737229 polymorphism had higher LDL cholesterol levels [135 mg/dL (110–148)] than those with homozygous *CC* [118 mg/dL (99–139)] (*p* < 0.003). The analysis of the *TRIB1* rs2954029 polymorphism showed that carriers of the *TT* genotype had increased glucose levels [97 mg/dL (87–118)] compared to carriers of the *AA* genotype [91 mg/dL (84–99)] (*p* < 0.002). In summary, our findings showed that, in Mexican Mestizos, the *AA* genotype of the *TRPS1* rs2737229 *A*/*C* SNP and the *TT* genotype of the *TRIB1* rs2954029 *A*/*T* polymorphism are associated with a higher risk of developing SA and elevated levels of glucose and LDL cholesterol.

## Simple Summary

Early detection of individuals at high risk of atherosclerosis is a priority worldwide. In this context, several genomic studies have suggested that polymorphic sites in the *TRPS1* and *TRIB1* genes are associated with the risk of cardiovascular disease and changes in plasma lipid concentrations. However, little is known about whether the incidence of subclinical atherosclerosis (SA) is associated with polymorphic sites in the *TRPS1* and *TRIB1* genes. In this study, we determined that the *TRIB1* rs2954029 *T*/*A* and *TRPS1* rs2737229 *A*/*C* polymorphic sites were associated with the risk of developing SA and with changes in glucose and LDL cholesterol levels, as previously observed in patients with clinical cardiovascular disease. Such an association suggests that these polymorphisms are involved in susceptibility to SA, possibly through increased LDL cholesterol and glucose levels, thereby conditioning the development of atherosclerotic plaque even in early stages before clinical manifestations.

## 1. Introduction

Dyslipidemia is characterized by elevated plasma lipid concentrations and is one of the most important risk factors for the development of atherosclerosis, including subclinical atherosclerosis (SA) [1,2,3,4]. Subclinical atherosclerosis (SA) is a condition in which coronary calcium deposits are detected without clinical evidence of cardiovascular disease. The Agatston score, derived from non-contrast computed tomography scans, is the standard method for quantifying coronary artery calcification (CAC). This score is a well-validated surrogate marker for diagnosing and stratifying the burden of SA [1,2,3,4]. Traditional cardiovascular risk factors, along with the patient’s genetics, contribute to the development of SA [4,5,6,7]. Thus, identifying genetic risk factors is relevant for preventing SA and its potential conversion to clinical atherosclerosis.

Among the potential genetic factors conditioning SA, previous studies have shown that the *TRPS1* and *TRIB1* genes [8,9,10,11,12,13] are associated with cardiovascular diseases (CVD) and, in particular, with changes in plasma lipid concentrations that may enhance the development of SA [8,9,10,11,12,13]. *TRPS1* and *TRIB1* are located on chromosome 8q23-24 and encode for the transcriptional repressor GATA-binding 1 (Trps1) and Tribbles pseudokinase 1 (Trib1) proteins [8]. However, the molecular mechanisms by which Trps1 and Trib1 influence the plasma lipid levels are poorly understood. In this context, *TRPS1* reduces hepatocyte nuclear factor 3-alpha (HNF-3A) (also known as forkhead box A1 (*FOXA1*) binding to the promoter region of *SCARB1*, leading to decreased expression of scavenger receptor B1 (SR-BI) [14]. This decrease can impair the transport of LDL and HDL cholesterol to the liver [15], inducing dyslipidemia. On the other hand, *TRIB1* has been associated with several diseases due to its role as a modulator of important biological processes, including those related to the plasma lipid profile [16,17]. Studies in mice demonstrated that when Trib1 was overexpressed, cholesterol and triglyceride levels decreased. In contrast, when *TRIB1* was removed (Trib1_LSKO), cholesterol and triglyceride levels increased, along with higher levels of *CCAAT* alpha enhancer binding protein (C/EBP-α) in the liver [18]. In addition, the interaction between the Trib1-Sin3A proteins and SAP18 induces the activation of the microsomal triglyceride transporter protein (MTTP), which is crucial for the assembly of apoB-containing particles, thereby enhancing hepatic lipogenesis [19,20,21].

Recent studies have shown that two single nucleotide polymorphisms (SNPs) (rs2980880 *T*/*C* and rs2954029 *T*/*A*) located in the intronic region of the *TRIB1* gene, as well as SNPs (rs231150 *A*/*T,* located in the downstream transcription region, and rs2737229 *A*/*C,* ubicated in the intronic region) of the *TRPS1* gene, play an important role in individuals’ susceptibility to developing coronary artery disease (CAD), myocardial infarction, hypercholesterolemia, acute coronary syndrome, and diabetes mellitus 2 (DM2) [8,9,10,11,12,13,22]. Therefore, we hypothesized that *TRPS1* and *TRIB1* polymorphisms are linked with the early development of SA through deregulation of plasma lipid levels. Thus, the main objective of this work was to evaluate whether the rs231150 *A*/*T*, rs2737229 *A*/*C*, rs2980880 *T*/*C*, and rs2954029 *T*/*A* SNPs are associated with susceptibility to developing SA and plasma lipid concentrations.

## 2. Materials and Methods

### 2.1. Participants

We recruited 1406 healthy Mexican mestizo participants without a familial predisposition to cardiovascular diseases (CVD) from the GEA Mexican study, recruited from June 2008 to January 2013 at the Instituto Nacional de Cardiología Ignacio Chávez [22]. The main inclusion criteria for the 1406 individuals were the absence of a personal or family history of CVD or of current or prior heart failure. Exclusion criteria included liver, renal, thyroid, and oncological diseases, as determined by clinical chemistry and medical exploration. The sample size was calculated for an unmatched case–control study, using an alpha error of 0.05 with a power of 80% (https://www.openepi.com/SampleSize/SSCC.htm; accessed 10 February 2026). According to this calculation, the required sample size was 640 individuals (320 patients with AS and 320 controls). All subjects had computed tomography to evaluate the CAC score [23]. Employing a CAC score exceeding 0 in the absence of clinical signs of CVD to delineate subclinical atherosclerosis (SA) [1,2,3,4], researchers identified 417 patients with SA. Meanwhile, 989 patients with a CAC score of 0 were included as controls. Subjects were ethnically matched and classified as Mexican mestizos if they and their ancestors (spanning at least three generations) were born in Mexico. The research protocol strictly followed the principles and ethical norms established in the Declaration of Helsinki. Moreover, all participants who consented to take part signed a letter of informed consent. The Ethics and Research Committees of our institution revised and approved the procedures and interventions performed in this study (project number 25-1519).

### 2.2. The Laboratory Performed an Evaluation

Plasma lipid profile and glucose levels were assessed spectrophotometrically in an automated analyzer using commercial kits from Randox Laboratories Ltd. (Kearneysville, WV, USA). The concentrations of high-density lipoprotein cholesterol (HDL-C) were assessed utilizing HDL cholesterol from SPINREACT, Girona, Spain. Low-density lipoprotein cholesterol (LDL-C) levels were calculated utilizing Friedewald’s technique for blood samples with triglyceride values below 400 mg/dL [24]. Dyslipidemia is characterized by a total cholesterol level exceeding 200 mg/dL, LDL-C level surpassing 130 mg/dL, HDL-C level below 40 mg/dL, or triglyceride levels exceeding 150 mg/dL [25]. Patients were classified as diabetic if their fasting glucose levels exceeded 125 mg/dL or if they were utilizing anti-diabetic medications [26]. Individuals having a systolic blood pressure of >130 mmHg and a diastolic blood pressure of >80 mmHg or receiving oral antihypertensive treatment were classified as hypertensive [27].

### 2.3. Genomic Analyses

DNA was isolated from peripheral blood cells with the QIAamp DNA Blood Mini Kit (QIAGEN, Hilden, Germany) and preserved at −80 °C until required. The SNPs in *TRPS1* (rs231150 *A*/*T*, rs2737229 *A*/*C*) and *TRIB1* (rs2980880 *T*/*C*, rs2954029 *T*/*A*) were identified using TaqMan assays on Real-Time PCR equipment, following the manufacturer’s cycling protocol. The sample was heated to 95 °C for 10 min, followed by 1 min at 60 °C. Subsequently, 40 cycles of heating to 95 °C for 15 s were conducted to denature the DNA strands, followed by cooling to 60 °C for 1 min to facilitate strand reannealing. To ensure genotyping quality, we genotyped 10% of the samples twice; the results were concordant in all cases. Supplementary Table S1 displays the chromosome position, base alteration, risk allele, and gene location of the SNPs.

### 2.4. Statistical Analysis

The Hardy–Weinberg equilibrium was assessed using the chi-squared test. A statistical analysis of the clinical and demographic data, as well as the association of the polymorphisms with AS patients, was performed with SPSS version 18.0 (SPSS, Chicago, IL, USA). The Shapiro–Francia test was used to assess the data distribution. According to this test, normally distributed variables were analyzed using Student’s *t*-test and expressed as mean ± standard deviation (SD). For non-normally distributed variables, the Mann–Whitney U test was used, and results are presented as median and interquartile [25th–75th]. Fisher’s exact test or chi-squared test was employed for categorical variables. To determine the association between the rs231150 *A*/*T*, rs2737229 *A*/*C*, rs2980880 *T*/*C*, and rs2954029 *T*/*A* polymorphisms and the susceptibility to developing SA, we used logistic regression analysis employing inheritance models (additive, codominant, dominant, over-dominant (heterozygous), and recessive) [28,29]. The logistic regression analysis was adjusted for cardiovascular risk factors (age, sex, body mass index (BMI), hypertension, smoking, and diabetes prevalence). The use of inheritance models provides a more nuanced understanding of the contributions of these SNPs, lipid profiles, and plasma glucose levels. This statistical procedure supports the interpretation that the observed associations between the genotype and plasma lipid levels, glucose, and other risk factors of SA are not merely a reflection of systemic metabolic noise, but a targeted genetic influence on the analyzed variables. The *p*-values were adjusted utilizing the Bonferroni correction. The findings are reported as OR (95% CI). The statistical power for this study was 0.80, according to OpenEpi version 3.01 (http://www.openepi.com/SampleSize/SSCC.htm; accessed 12 November 2024). *p*-values less than 0.05 were considered statistically significant. Haplotype analysis was conducted using Haploview version 4.1 [30]. Haploview utilizes data from the international HapMap project and a standardized methodology to estimate haplotypes, examining the potential co-inheritance of certain gene variants due to their proximity on the chromosome.

To evaluate the association between the polymorphisms rs231150 *A*/*T,* rs2737229 *A*/*C,* rs2980880 *T*/*C,* and rs2954029 *T*/*A* and the phenotype (i.e., plasma lipid and glucose concentrations), individuals were categorized by SNP genotype. Once categorized, the possible statistical relationships between genotypes and lipid profile levels (total cholesterol, HDL-C, LDL-C, and triglycerides) and glucose levels were determined. For this analysis, we used the Kruskal–Wallis test, followed by a post hoc Mann–Whitney U test.

## 3. Results

### 3.1. Anthropometric and Biochemical Parameters

The clinical and demographic characteristics of the participants are summarized in Table 1. Significant disparities in cardiovascular risk factors were observed between individuals with subclinical atherosclerosis (SA) and the healthy control group. Specifically, the SA group had a higher age and a higher proportion of male participants, as well as a higher prevalence of hypertension compared to controls (*p* < 0.001). In addition, systolic and diastolic blood pressure were higher in patients with AS than in control individuals (*p* < 0.001).

Regarding metabolic parameters, patients with SA exhibited significantly higher fasting glucose, triglycerides, and LDL cholesterol (LDL-C) levels than those in the control group (*p* < 0.05). On the other hand, HDL cholesterol (HDL-C) concentrations were notably lower in patients with SA than in the healthy group (*p* < 0.05). No statistically significant differences were found between the groups concerning body mass index (BMI), total cholesterol, and smoking status.

### 3.2. Association of TRPS1 and TRIB1 Polymorphisms with SA

The distribution of the evaluated *TRPS1* and *TRIB1* polymorphisms conformed to the Hardy–Weinberg equilibrium in both study groups (*p* > 0.05). A comparative analysis revealed distinct differences in the allelic and genotypic frequencies of the rs2737229 *A*/*C* and rs2954029 *T*/*A* SNPs between SA cases and controls (*p* < 0.05) (Supplementary Table 2). By applying multiple inheritance models (Table 2), it was determined that the minor allele *A* of the *TRPS1* rs2737229 *A*/*C* polymorphism significantly is associated with an increased risk of developing SA, according with following inheritance models: codominant (OR = 1.61, 95% CI = 1.10–2.36, pC = 0.048), recessive (OR = 1.42, 95% CI = 1.02–1.99, pC = 0.039), and additive (OR = 1.26, 95% CI = 1.05–1.53, pC = 0.015) (Table 2). The analysis of the *TRIB1* rs2954029 *T*/*A* SNP indicated that individuals with the *T* allele are associated with an increased risk of developing SA, according to the codominant (OR = 1.63, 95% CI = 1.10–2.43, pC = 0.033) and recessive (OR = 1.64, 95% CI = 1.13–2.37, pC = 0.009) models (Table 2).

### 3.3. Haplotype Analysis

The haplotype analysis results for both genes are presented in Table 3. Analysis of the *TRPS1* gene showed that the rs231150 *A*/*T* and rs2737229 *A*/*C* polymorphisms were not in linkage disequilibrium. Nonetheless, our findings revealed two haplotypic combinations between rs231150 *A*/*T* and rs2737229 *A*/*C* polymorphisms associated with SA (Table 3). The “*TA*” combination was associated with an elevated risk of developing SA (OR = 1.20, *p*C = 0.034), whereas the “*AC*” combination was associated with a low risk of developing SA. In the case of the *TRIB1* gene, the rs2980880 *C*/*T* and rs2954029 *A*/*T* polymorphisms showed strong linkage disequilibrium (D’ = 0.93). However, the haplotype distribution was similar and not significant between patients with SA and the control group (Table 3).

### 3.4. Genotype–Phenotype Relationship: Glucose and Lipid Concentrations

**Figure 1:**
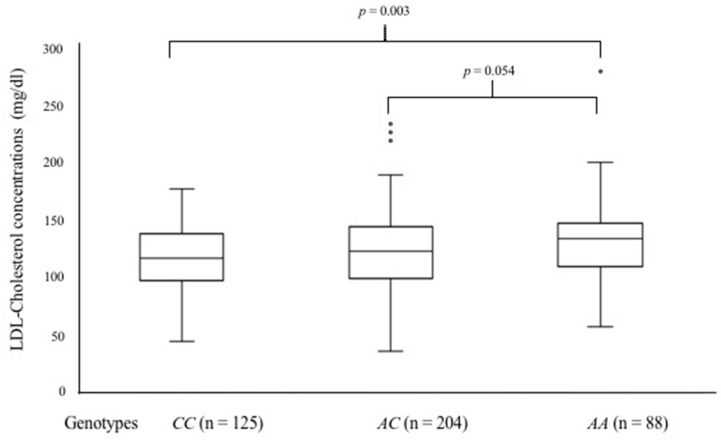
LDL cholesterol concentrations in patients with SA stratified by genotypes at the rs2737229 polymorphism in the *TRPS1* gene. Data are presented as median [interquartile range]. Group comparisons were assessed using the Kruskal–Wallis test, followed by a post hoc Mann–Whitney U test. Accordingly, *p*-values less than 0.05 were considered statistically significant.

**Figure 2:**
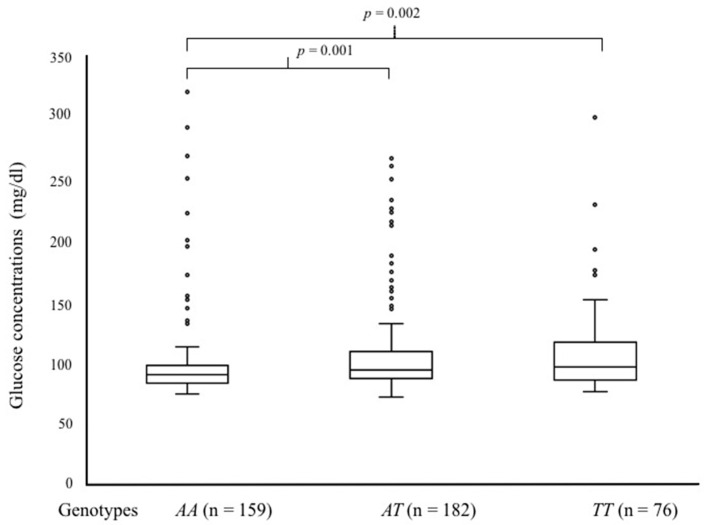
Glucose levels in 417 patients with SA, categorized by genotypes of the *TRIB1* rs2954029 *T*/*A* polymorphism. Data are presented as median [interquartile range]. Group comparisons were assessed using the Kruskal–Wallis test, followed by a post hoc Mann–Whitney U test. Accordingly, *p*-values less than 0.05 were considered statistically significant.

Patients were grouped by *TRPS1* rs2737229 *A*/*C* and *TRIB1* rs2954029 *A*/*T* genotypes, and the biochemical parameters (glucose, total cholesterol, HDL cholesterol, LDL cholesterol, and triglycerides) were compared between groups. Interestingly, two main risk factors for SA differed in this analysis: LDL cholesterol and plasma glucose levels (Figure 1 and Figure 2, respectively). The homozygous *TRPS1* rs2737229 *AA* individuals exhibited higher LDL-C plasma levels [135 mg/dL (110–148)] than homozygous *CC* individuals [118 mg/dL (99–139)] (*p* < 0.003), as shown in Figure 1. This analysis indicates that carriers of the *TRPS1* rs2737229 *A* allele are more likely to have higher plasma LDL-C levels. Furthermore, the *TRIB1* rs2954029 *T*/*A* polymorphism revealed that individuals carrying the *T* allele (*TT* and *AT* genotypes) exhibited elevated glucose plasma levels [97 mg/dL (87–118) and 95 mg/dL (88–110), respectively] compared to homozygous *AA* subjects [91 mg/dL (84–99)], as shown in Figure 2.

## 4. Discussion

Certain genetic variants in the transcriptional repressor GATA binding 1 (*TRPS1*) and Tribbles pseudokinase 1 (*TRIB1*) genes have been controversially linked to susceptibility to coronary heart disease (CHD) and plasma lipid profiles [8,9,10,11,12,13,22]. Consequently, we assessed the associations of the *TRPS1* (rs231150 *A*/*T*, rs2737229 *A*/*C*) and *TRIB1* (rs2980880 *T*/*C*, rs2954029 *T*/*A*) SNPs with the risk of developing SA, as well as their relationships with plasma lipid concentrations. Our data indicate that the minor alleles (rs2737229 *A* and rs2954029 *T*) are associated with an increased risk of developing SA. To the best of our knowledge, our study is the first to elucidate the correlation between these SNPs and SA; in particular, the relationship between these SNPs and the occurrence of SA in other groups has not been documented. In our study, the *TRPS1* rs2737229 *A* allele was associated with an increased risk of developing SA. In line with our data, Vargas-Alarcon et al. reported that the rs2737229 *A* allele was associated with an increased risk of developing acute coronary syndrome (ACS) and with lower triglyceride levels [22]. Conversely, some studies have indicated that this SNP is not associated with the risk of developing CHD across various populations, whereas it is linked to alterations in plasma lipid levels [8,13,31]. In this regard, we reported that individuals with the rs2737229 *AA* genotype had higher levels of plasma LDL-C than the *CC* homozygous individuals. In turn, LDLs are lipoproteins directly associated with the development of atheroma [32], suggesting that the association between the rs2737229 *AA* genotype and susceptibility to SA may be mediated by higher levels of these lipoproteins in carriers of the *AA* genotype. However, this relationship is only statistical, and the demonstration of a causal relationship between rs2737229 *AA*, as well as TRIB1 rs2954029 *TT*, genotypes and SA merits to be investigated. Concerning the *TRIB1* rs2954029 *A*/*T* genetic variant, our data showed that the *T* allele or *TT* genotype was associated with SA and, unexpectedly, with elevated glucose levels in our cohort. Recent studies have shown that allele *T* is associated with dyslipidemias, particularly variations in plasma HDL-C levels, as well as with ACS and CHD [8,18,22,33,34,35]. Nonetheless, previous investigations indicated that the *A* allele affects total cholesterol, triglycerides, and LDL-C levels, and is correlated with an elevated risk of developing CHD and ischemic stroke [13,32,36]. According to the literature and our data, the role of these polymorphisms in CHD, as well as in plasma lipid concentrations, remains controversial. This phenomenon may be attributed to the ethnic background of the studied populations; allele frequencies for rs231150 *A*, rs2737229 *A*, rs2980880 *C,* and rs2954029 *T* have been reported to be lower in the Mexican population than in Caucasian, Asian, and African populations [22]. Therefore, the effect of these SNPs on SA should be explored further in multicentric studies that include individuals of diverse ethnic origins. Ultimately, haplotype analysis indicated that the “*TA*” haplotype derived from *TRPS1* (rs231150 *A*/*T* and rs2737229 *A*/*C*) was linked to an increased risk of developing SA. The “*TT*” haplotype, comprising *TRIB1* (rs2980880 *T*/*C* and rs2954029 *T*/*A*), was more prevalent in patients with SA (38.7%) compared to controls (35.5%). Nonetheless, this difference did not reach statistical significance (*pC* = 0.058). Nonetheless, these data suggest that this haplotype may be considered in future studies to assess the risk of developing SA. Collectively, our results endorse the notion that rs2737229 *A/C* and rs2954029 *T/A* polymorphisms are associated with the risk of developing SA, as has been reported in cardiovascular diseases such as CAD, myocardial infarction, hypercholesterolemia, and acute coronary syndrome, and in metabolic diseases such as DM2 [8,9,10,11,12,13,22].

Moreover, when patients with SA were categorized by genotype, our findings indicated that *TRPS1* (rs2737229 *A*/*C*) and *TRIB1* (rs2954029 *T*/*A*) SNPs significantly influence plasma LDL-C and glucose levels. These findings showed that the *TT* and *TA* genotypes of the rs2954029 SNP are associated with increased glucose levels. On the other hand, individuals with the rs2737229 *AA* genotype had elevated LDL-C levels. Experimental studies indicate that Trib1 overexpression reduces cholesterol and triglyceride levels in mouse hepatocytes. In contrast, reduced expression of this gene correlates with elevated cholesterol, triglycerides, and C/EBP-alpha levels, the latter of which play a significant role in lipid metabolism [37]. Additionally, the *TRIB1* gene plays an important role in the assembly of VLDL particles by activating MTTP, which binds to SAP18 (a subunit of the Sin3A-HDAC transcriptional repressor complex), thereby increasing plasma lipid levels [19,20,21]. Lastly, the Trib1 protein modulates many biological processes, including the levels of C/EBP-alpha and ChREBP proteins in the liver [18]. These transcription factors regulate several genes in glucose metabolism. Therefore, we suggest that the *T* allele of the *TRIB1* rs2954029 *T*/*A* polymorphism may affect gene expression and, consequently, glucose plasma levels in an indirect manner mediated by C/EBP-alpha and ChREBP proteins. However, further studies are necessary to elucidate the mechanisms underlying the relationship between *TRIB1* and plasma glucose levels. Finally, *TRPS1* encodes a repressor GATA-sequence-binding transcription factor that regulates the expression of target genes [38,39]. In this context, *SCARB1* is recognized as one of the genes regulated by *TRPS1*, thus decreasing the scavenger receptor B1 (SR-BI) expression [14]. Such a decrease may be related to an impaired *p*-value, as observed in our study. Therefore, we speculate that the *TRPS1* rs2737229 *A* allele may contribute to an enhanced *TRPS1* gene expression, thereby affecting LDL cholesterol catabolism. As experimental and genome association studies, including our results, have yielded different findings regarding the involvement of these genes in atherosclerosis and plasma lipid levels, we advocate for the necessity of bioinformatics prediction studies and functional studies to elucidate the contribution of polymorphic sites of *TRPS1* and *TRIB1* to the expression of their coding proteins, as well as the genuine roles of these polymorphisms in genetic susceptibility to SA.

### Limitations of the Study

The number of patients with SA was lower than the number of controls. The sex distribution in SA individuals and controls also differed; this difference was treated as a confounding variable and accounted for in the analyses to interpret the study findings. The findings of this study did not elucidate the molecular mechanism by which *TRPS1* and *TRIB1* SNPs influence plasma lipid levels; it also demonstrated a statistical association between cardiovascular risk factors and the genetic predisposition to developing SA, as well as the alleles potentially associated with plasma lipid levels.

## 5. Conclusions

The results obtained in this study suggest that the *TRPS1* rs2737229 *A*/*C* and *TRIB1* rs2954029 *T*/*A* polymorphisms were associated with a higher risk of developing SA and with elevated glucose and LDL cholesterol levels in our population.

## Figures and Tables

**Table 1:** The parameters of the studied individuals.

Characteristics		SA Patients (n = 417)	Healthy Controls (n = 989)	*p*-Value
Age (years)		58.3 ± 8.7	51.0 ± 8.85	<0.001
BMI (kg/m^2^)		28.6 ± 3.9	28.2 ± 4.07	NS
Gender n (%)	Male	311 (74.5)	398 (40.2)	<0.001
	Female	106 (25.4)	591 (59.7)	<0.001
Hypertension, n (%)	Yes	191 (45.8)	183 (18.5)	<0.001
Smoking, n (%)	Yes	97 (23.2)	219 (21.9)	NS
DM2, n (%)	Yes	61 (14.6)	---	---
Glucose (mg/dL)		94 [86–104]	89 [83–95]	<0.001
Systolic blood pressure (mmHg)		121 [111–133]	112 [103–122]	<0.001
Diastolic blood pressure (mmHg)		74 [68–81]	70 [65–76]	<0.001
Total cholesterol (mg/dL)		196 [168–219]	190 [167–211]	NS
HDL-C (mg/dL)		43 [36–51]	45 [36–55]	0.002
LDL-C (mg/dL)		123 [101–145]	116 [96–134]	0.018
Triglycerides (mg/dL)		154 [118–204]	144 [107–201]	0.017

Continuous variables are expressed as mean ± SD or median [interquartile range], while categorical variables are shown as absolute frequencies. Intergroup differences were analyzed via Student’s *t*-test, the Mann–Whitney U test, or the chi-square test. SA = subclinical atherosclerosis, DM2 = diabetes mellitus 2, NS = not significant, and BMI = body mass index.

**Table 2:** Evaluation of *TRIB1* and *TRPS1* variants as risk factors for SA across different genetic inheritance patterns.

SNP (rsID) * Inheritance Model	Genotype	Patients with SA 417 (n (%))	Controls 989 (n(%))	OR (95% CI)	*p*C
*TRPS1* rs2737229					
Codominant	*CC* *AC* *AA*	125 (30.0) 204 (48.9) 88 (21.1)	348 (35.2) 485 (49.0) 156 (15.8)	1.61 (1.10–2.36)	0.048
Dominant	*CC* *AC* + *AA*	125 (30.0) 292 (70.0)	348 (35.2) 641 (64.8)	1.32 (0.99–1.74)	0.055
Recessive	*CC* + *AC* *AA*	329 (78.9) 88 (21.1)	833 (84.2) 156 (15.8)	1.42 (1.02–1.99)	0.039
Over-dominant	*CC* + *AA* *AC*	213 (51.1) 204 (48.9)	504 (51.0) 485 (49.0)	1.03 (0.79–1.33)	0.832
Additive	--------	---------	-----------	1.26 (1.05–1.53)	0.015
*TRIB1* rs2954029					
Codominant	*AA* *AT* *TT*	159 (38.4) 182 (43.6) 76 (18.2)	408 (41.2) 456 (46.1) 125 (12.6)	1.63 (1.10–2.43)	0.033
Dominant	*AA* *AT* + *TT*	159 (38.1) 258 (61.9)	408 (41.2) 581 (58.8)	1.13 (0.86–1.49)	0.391
Recessive	*AA* + *AT* *TT*	341 (81.8) 76 (18.2)	864 (87.4) 125 (12.6)	1.64 (1.13–2.37)	0.009
Over-dominant	*AA* + *TT* *AT*	235 (56.4) 182 (43.6)	533 (53.9) 456 (46.1)	0.87 (0.66–1.14)	0.298
Additive	--------	---------	-----------	1.21 (1.00–1.47)	0.051

SNP = single nucleotide polymorphism, SA = subclinical atherosclerosis, OR = odds ratio, CI = confidence interval, and pC = *p*-value corrected by the Bonferroni test. * The inheritance models (additive, codominant, dominant, over-dominant (heterozygous), and recessive) were designed based on the minor allele. In addition, all models were analyzed using logistic regression, including gender, age, BMI, smoking status, and incidence of hypertension and diabetes.

**Table 3:** An analysis of *TRPS1* and *TRIB1* haplotype profiles across the study population.

* Polymorphic Site	SA Patients n = 417	Controls n= 989	OR	95% CI	*pC*
Block Haplotype	Hf (%)	Hf (%)			
*T C*	0.369	0.392	0.90	0.76–1.07	0.119
*A A*	0.235	0.212	1.14	0.93–1.38	0.092
*T A*	0.220	0.190	1.20	0.99–1.46	0.034
*A C*	0.176	0.205	0.82	0.67–1.01	0.038
Block Haplotype	Hf (%)	Hf (%)			*p*
*T A*	0.375	0.392	0.93	0.78–1.09	0.198
*T T*	0.387	0.355	1.14	0.97–1.35	0.058
*C A*	0.225	0.251	0.86	0.71–1.05	0.066

SA = subclinical atherosclerosis, Hf = haplotype frequency, *p*C = *p*-value corrected via the Bonferroni test. * Polymorphism order and haplotype configurations correspond to their physical locations on chromosome 8: region 8q23.3 (rs231150 *A*/*T*; rs2737229 *A*/*C*) and region 8q24.13 (rs2980880 *C*/*T*; rs2954029 *A*/*T*).

## Data Availability

The dataset used in this study is available upon reasonable request to the corresponding author.

## Supplementary Materials

The following supporting information can be downloaded at https://www.mdpi.com/article/10.3390/biology15070580/s1. Table S1: Information about the polymorphisms studied; Table S2: Allelic and genotypic frequency of the rs231150 *A*/*T*, rs2737229 *A*/*C,* rs2980880 *T*/*C,* and rs2954029 *T*/*A* polymorphisms in SA patients and controls.
