# Supplementary material for: Association of TRPS1 rs2737229 and TRIB1 rs2954029 Genetic Polymorphisms with Subclinical Atherosclerosis, LDL Cholesterol, and Glucose Levels
Source: Biology (Basel). 2026 Apr 5;15(7):580. doi: 10.3390/biology15070580 (PMC13072123; doi:10.3390/biology15070580)
Supplement: Supplementary file 1 [file biology-15-00580-s001.zip › biology-4177649-supplementary.pdf]

Supplementary Information

Supplementary Table S1. Information about the polymorphisms studied.

Gene symbol	SNP (rsID-number)[a,b]	Chromosome	Chromosome position	Allele minor	Location in gene
TRPS1	rs231150	8q23.3	115408099	*A*	Downstream Transcript Variant
TRPS1	rs2737229	8q23.3	115636338	*A*	Intron Variant
TRIB1	rs2980880	8q24.13	125468730	*C*	Intron Variant
TRIB1	rs2954029	8q24.13	125478730	*T*	Intron Variant

[a] Single Nucleotide Polymorphisms (rsID-number) in database dbSNP; [b] Given name according to NCBI, Current Build 156 (released September 21, 2024) https://www-ncbi-nlm-nih-gov.pbidi.unam.mx:2443/snp/?term=rs231150.

Supplementary Table S2. Allelic and genotypic frequency of the rs231150 *A/T*, rs2737229 *A/C,* rs2980880 *T/C,* and rs2954029 *T/A* polymorphisms in SA patients and controls.

SNP (rsID-number)	SA n=417, n (%)	Controls n=989. n (%)	*p
rs231150 *A/T*			
Allele			
T	491 (58.8)	1152 (58.2)	
A	343 (41.1)	826 (41.7)	NS
Genotype			
TT	145 (34.8)	326 (33.0)	
AT	201 (48.2)	500 (50.6)	
AA	71 (17.0)	163 (16.5)	NS
rs2737229 *A/C*			
Allele			
C	454 (54.4)	1181 (59.7)	
A	380 (45.5)	797 (40.2)	0.004
Genotype			
CC	125 (30.0)	348 (35.2)	
AC	204 (48.9)	485 (49.0)	
AA	88 (21.1)	156 (15.8)	0.007
rs2980880 *C/T*			
Allele			
T	635 (76.1)	1478 (74.7)	
C	199 (23.8)	500 (25.2)	NS
Genotype			
TT	245 (58.8)	554 (56.0)	
TC	145 (34.8)	370 (37.4)	
CC	27 (6.5)	65 (6.6)	NS
rs2954029 *T/A*			
Allele			
A	500 (59.9)	1272 (64.3)	
T	334 (40.0)	706 (35.6)	0.014
Genotype			
AA	159 (38.1)	408 (41.2)	
AT	182 (43.6)	456 (46.1)	
TT	76 (18.2)	125 (12.6)	0.003

SNP, Single nucleotide polymorphism, SA, Subclinical atherosclerosis Data are shown as n and frequency expressed as percentage. *chi-square test. NS: No significant
